# *Streptococcus pneumoniae* from Palestinian Nasopharyngeal Carriers: Serotype Distribution and Antimicrobial Resistance

**DOI:** 10.1371/journal.pone.0082047

**Published:** 2013-12-10

**Authors:** Abedelmajeed Nasereddin, Issa Shtayeh, Asad Ramlawi, Nisreen Salman, Ibrahim Salem, Ziad Abdeen

**Affiliations:** 1 Al-Quds Nutrition and Health Research Institute, Faculty of Medicine, Al-Quds University, Abu-Deis, The West Bank, Palestine; 2 Central Public Health Laboratory, Palestinian Ministry of Health, Ramallah, Palestine; Rockefeller University, United States of America

## Abstract

Infections of *Streptococcus pneumoniae* in children can be prevented by vaccination; left untreated, they cause high morbidity and fatalities. This study aimed at determining the nasopharyngeal carrier rates, serotype distribution and antimicrobial resistance patterns of *S. pneumoniae* in healthy Palestinian children under age two prior to the full introduction of the pneumococcal 7-valent conjugate vaccine (PCV7), which was originally introduced into Palestine in a pilot trial in September, 2010. In a cross sectional study, nasopharyngeal specimens were collected from 397 healthy children from different Palestinian districts between the beginning of November 2012 to the end of January 2013. Samples were inoculated into blood agar and suspected colonies were examined by amplifying the pneumococcal-specific autolysin gene using a real-time PCR. Serotypes were identified by a PCR that incorporated different sets of specific primers. Antimicrobial susceptibility was measured by disk diffusion and MIC methods. The resulting carrier rate of *Streptococcus pneumoniae* was 55.7% (221/397). The main serotypes were PCV7 serotypes 19F (12.2%), 23F (9.0%), 6B (8.6%) and 14 (4%) and PCV13 serotypes 6A (13.6%) and 19A (4.1%). Notably, serotype 6A, not included in the pilot trial (PCV7) vaccine, was the most prevalent. Resistance to more than two drugs was observed for bacteria from 34.1% of the children (72/211) while 22.3% (47/211) carried bacteria were susceptible to all tested antibiotics. All the isolates were sensitive to cefotaxime and vancomycin.

Any or all of these might impinge on the type and efficacy of the pneumococcal conjugate vaccines and antibiotics to be used for prevention and treatment of pneumococcal disease in the country.

## Introduction

Pneumococcal infection caused by *Streptococcus pneumoniae* is a major contributor to morbidity and the main cause of deaths preventable by vaccination in children under age five worldwide [1], [2]. The World Health Organization (WHO) estimates that more than one million children die of pneumococcal disease in developing countries every year [3]. The range of pneumococcal infections is wide and often preceded by an asymptomatic carrier state, in the nasopharynx, mainly in pre-school children [4]. The presence of *S. pneumoniae* in the nasopharynx of healthy children is indicative of the strains circulating and causing infections in the community, and has often been reported as being a precursor to waves of invasive disease and a major factor in the spread of infection [5]–[7].

Currently, more than 90 distinct serotypes of *S. pneumoniae* have been described, based on the structure of the capsular polysaccharides, which are considered to be the major virulence factors [8]–[10]. Only some of the serotypes cause disease and, of those that do, some have a greater capacity to invade and cause bacteraemias. Others are more frequently associated with respiratory tract disease without a bacteraemia, while still others remain limited to the nasopharynx, bacteria lacking a polysaccharide capsule rarely cause invasive disease [11].

Prevention of infections caused by *S. pneumoniae* and their spread in young children is such an important goal of effective vaccination that new vaccines have been developed to achieve this. In 2007, the WHO recommended that pneumococcal conjugate vaccine 7 (PCV7), which contains the polysaccharides of the serotypes 4, 6B, 9V, 14, 18C, 19F and 23F, be used in national immunization programs (NIP) [3]. Two more PCVs were recently introduced: PCV10, which contains the polysaccharides of the serotypes 1, 5, and 7F in addition to the serotypes of PCV7, and PCV13, which contains the polysaccharides of the serotypes 3, 6A, and 19A in addition to the serotypes of PCV10 [12], [13]. PCVs of higher valence provide broader serotype coverage [14], [15]. The use of PCVs in young children creates herd immunity by reducing transmission and, thus, circulation of the bacterial serotypes opposed by the vaccine, leading to a decrease in invasive pneumococcal disease (IPD) in the population [16], [17].

Treatment of pneumococcal infections is becoming more difficult owing to the emergence of antibiotic-resistant pneumococci. Studies on serotype distribution and their antibiotic sensitivities are necessary for planning rational national strategies for preventing and treating IPD [6]. Surveillance of pneumococcal diseases according to bacterial serotypes is essential, to learn about the current serotype distribution and to observe the efficacy of PCVs by following the dynamics of bacterial serotypes in the population, following the introduction of vaccination. Several studies have reported regional and temporal changes in the distribution of bacterial serotypes after the application of PCVs [18], [19].

Kattan et al. [20] described the distribution of invasive *S. pneumoniae* from two Palestinian hospitals where bacteria of serotypes 6, 14, 1, and 9V predominated. The serotypes within serogroup 6 were not determined, however, it is important to identify the serotypes within serogroups such as serogroup 6 as serotype 6B polysaccharide is in PCV7 while serotypes 6A and 6C are not.

Following an introductory trial of the PCV7 vaccine in a pilot study in the Hebron, Bethlehem, Jericho and Tubas districts in September 2010, this study was done to determine the rate of nasopharyngeal pneumococcal infection in healthy children, identify the serotypes of the circulating strains of *S. pneumoniae* and determine their antibiotic susceptibilities.

## Materials and Methods

In this cross-sectional study, 197 female and 200 male healthy children more than two years old, attending the West Bank primary health care centers was examined. The children were selected according to their vaccination cards and distribution in different regions per district such that the number of participants per district was proportional to the size of the district population. Health workers contacted parents using the phone number inscribed on vaccination cards. The study was conducted from November 2012 through January 2013. After obtaining informed parental written consent, members of the study team examined each child and documented relevant data on a pre-designed questionnaire that requested the age, gender, district, location of residence, and whether the child had been vaccinated with PCV7 or not, and, if vaccinated, giving the number of doses. The Ethics Research Committee of Al-Quds University and Palestinian Ministry of Health in Ramallah approved all the study activities, including the written informed consent. Nasopharyngeal specimens were taken in the health care centers by a well-trained physician, who used extra-thin flexible flocked swabs, which were immediately transferred into Amies transport media tubes (Copan Diagnostics Inc, Murrieta, CA, USA) that were transported to the Central Public Health Laboratory (CPHL) in Ramallah within 2 h for the isolation, identification, serotyping and in vitro testing of antimicrobial susceptibility of *S. pneumoniae*.

### Pneumococcal isolate detection and identification

On arrival at the CPHL, swabs were streaked onto plates containing 5% sheep blood and 5 µg/ml gentamicin (Fluka). Plates were incubated for 18 to 24 h at 37°C in candle jars. Potential colonies of *S. pneumoniae* were carefully selected by colonial morphology and alpha-hemolysis, and confirmed by Gram staining and susceptibility to optochin (Himedia Laboratories Pvt. Ltd, India) (inhibition zone >14 mm). Samples were also examined with a Real Time PCR-based method for the pneumococcal-specific autolysin gene (lytA), of which the primer and probe sequences were as described by McAvin et al. [21].

The same nasopharyngeal flocked swabs were also inoculated into cryotubes containing 1.0 ml of skim milk, tryptone, glucose, glycerol (STGG) transport medium. To store isolates, each recovered pneumococcal isolate was sub-cultured on blood agar. The single colonies were then removed using sterile cotton swabs and, put into labelled cryotubes containing 1.0 ml STGG medium, and stored at −70°C until tested for serotype and antimicrobial susceptibility. Cultures were also lyophilized in skimmed milk and stored at −70°C.

### In vitro susceptibility testing

Testing the susceptibility of the strains of *S. pneumoniae* to penicillin (oxacillin) (1 µg), erythromycin (15 µg), trimethoprim–sulfamethoxazole (25 µg) and vancomycin (30 µg) (Oxoid Ltd, UK) was done according to Kirby Bauer, using a disk diffusion method on Mueller Hinton agar enriched with 5% sheep blood. Susceptibility to penicillin was also determined by E-test Antimicrobial Minimum Inhibitory Concentrations (MICs) as it was for cefotaxime. MICs and Disk Diffusion breakpoints used in this study were interpreted according to Clinical Laboratory Standards Institute (CLSI) guidelines [22]. All isolates of *S. pneumoniae* suspected of being penicillin resistant (oxacillin susceptibility: disk diffusion method <20 mm) were tested further for penicillin MICs by the E-test strip method according to the manufacturer's instructions (Oxoid). Penicillin G and Cefotaxime strains of *S. pneumoniae* were defined as isolates with a MIC ≥2 µg/ml as resistant, 0.12 µg/ml as intermediate resistance, and ≤0.06 µg/ml as susceptible.

### DNA extraction

Streptococcal DNA was extracted from the cultured bacteria using the CDC protocol (http://www.cdc.gov/ncidod/biotech/strep/pcr.htm).

#### Real-time PCR for *lytA*

The real-time PCR assay was carried out on a final reaction volume of 25 µl and performed using Eurogentec qPCR Master Mix (Eurogentec, Seraing, Belgium), containing low ROX as a passive reference, according to the manufacturer instructions, and 5 µl of DNA sample was used in each reaction. The primers and probe concentrations were 0.2 µM for each one. No template control (NTC) and *S. pneumoniae*-positive DNA control were included in each run. Amplification was done in the 7500 Real Time PCR system (Applied Biosystems) under the following PCR conditions: 95°C for 10 min, followed by 40 cycles of 95°C for 15 s and 60°C for 1 min.

### Capsular Serotyping

Serotyping of the isolates of *S. pneumoniae* was performed by a sequential multiplex PCR assay, using a set of primers targeting different serotype specific sequences as described by Pai et al [23]. The serotypes tested included 1, 3, 4, 5, 6A/B/C/D, 7C/7B/40, 7F/7A, 9V/9A, 9N/9L, 10A, 11A/11D, 12F/12A/44/46, 13, 14, 15A/15F, 15B/15C, 18 (A/B/C/F), 19A, 19F, 20, 21, 22F/22A, 23A, 23F, 24(A/B/F), 33F/33A/37, 34, 35B, 35F/47F, 38/25F/25A. All other serotypes were classified as nontypeable.

### Differentiation of the serogroup subtypes 6A/C and 6B of *S. pneumoniae*

Forty-nine isolates identified by the sequential multiplex PCR assay of *S. pneumoniae* belonged to the serogroup 6. It was important to identify the subtype 6A/C of the serogroup 6 since it is not included in the PCV7 vaccine. In addition, the published method for differentiating serogroup 6 subtypes requires a laborious and sophisticated effort so we introduced the restriction fragment length polymorphism (RFLP) method to distinguish serotype 6A/C from serotype B. The PCR method described by Jin et al. [24] to reveal sub-serotype specificity, using G584A polymorphism as a target, did not expose the specificity they described in our hands.

The universal reverse primer (wciP-r) of Jin et al. [24] was used. The new forward primer (ForSeq) given below was designed to amplify the region covering the entire single nucleotide polymorphism (SNP) and was used for RFLP analysis. Twenty-eight PCR-amplification samples of serogroup 6 were sent for sequencing using the wciP-r primer [24] and also using our own newly designed primer ForSeq: 5′ TGG GGA TTG AAT TAC CGA AC).

PCR amplification of the 28 samples was as follows: The final reaction volume of 50 µl contained DreamTaq Green PCR Master Mix (Thermo Scientific), 0.25 µM of each primer, and 5 µl of DNA template. Thermocycler conditions for amplification were: 95°C for 15 min, 35 cycles of 94°C for 30 s, 62°C for 60 s, and 72°C for 60 s, and a final extension of 72°C for 10 min followed by a hold at 4°C. By Blast analysis (http://blast.ncbi.nlm.nih.gov/) and multiple sequence alignments, 25 samples aligned with serotype 6A, 3 to serotype 6B, and none to serotype 6C. Reference sequences used were: serotype 6A (GenBank accession no. JF911497.1), 6B (GenBank accession no. JF911507.1), and 6C (GenBank accession no. JF911510.1). In addition to SNP G584A of Jin et al. [24], we noticed another SNP (G574T) that could be used to differentiate the 6A/C from the 6B serotype. By using the NEB cutter program (http://tools.neb.com/NEBcutter2/index.php), we mapped the restriction site of the enzyme BsrI (New England Biolabs), and showed distinct digestion patterns for the two serotypes: three bands aligning with the molecular weights for 145, 69 and 59 bp for 6A/C serotype: two bands aligning with 214 and 59 bp for 6B serotype.

For RFLP analysis, 20 µl of each PCR product was digested with BsrI for 4 h at 65°C. DNA fragments were resolved by electrophoresis on a 2% agarose gel.

**Figure 1:**
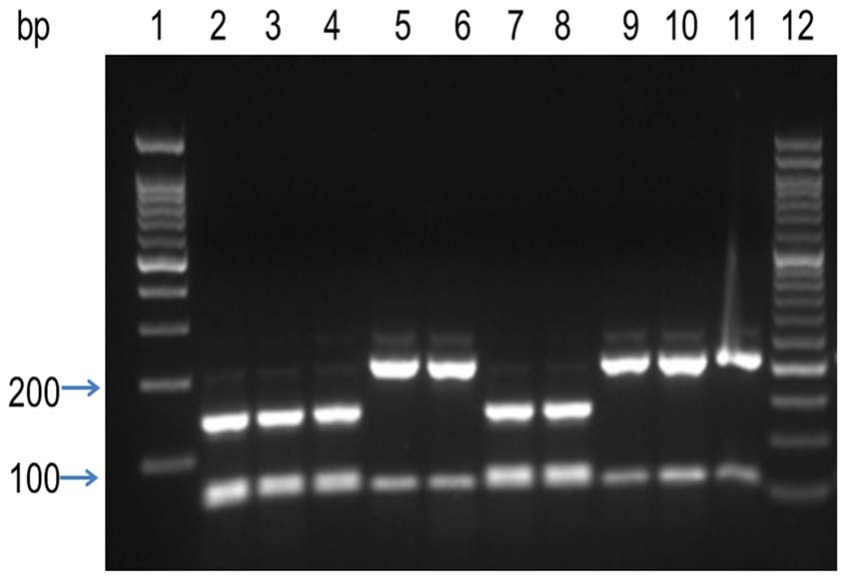
A representative DNA electrophoresis gel showing the RFLP patterns of the serotypes 6A/C and 6B after BsrI digestion of the amplified cps amplicon (273 bp) for isolates of *S. pneumonia* of the serogroup 6. DNA was visualized in 2% agarose gel: lane 1 100 bp ladder; lane 12 50 bp ladder. Lanes 2–4 and 7–8 *S. pneumonia* serotype 6A/C patterns (yielding fragments of 145, 69 and 59 bp, with both smaller fragments showing as one thick band owing to low separation). Lanes 5–6 and 9–11 *S. pneumonia* serotype 6B (yielding fragments of 214 and 59 bp).

In the case of serotype 6A/C, the amplicons digested by BsrI gave the 145 bp band, while the lower molecular weight bands 69 and 59 bp were seen as one band on the gel (Figure 1). In the case of serotype 6B, the bands of 214 and 59 bp, were clearly separated. Each DNA sample was analyzed at least in duplicate against serotype 6A and 6B positive controls as revealed by sequence analysis. The primers used for genotyping by PCR-RFLP of the serotype 6A/C and 6B SNPs were the same as those used for amplicon sequencing.

## Results

### Study Participation

Nasopharyngeal swabs were taken from 397 healthy children aged 1 to 23 months; mean age was 11 months. 248 (62.5%) of the children were less than 1 years old, 149 (37.5%) were between 1 and 2 years old. Gender distribution (Male: Female) was 1∶1. Vaccine PCV7 (containing serotypes: 4, 6B, 9V, 14, 18C, 19F, and 23F) was introduced in Palestine as a pilot trial since September 2010 in the following districts: Hebron, Bethlehem, Jericho and Tubas. One (0.3%) child received one dose of vaccine during the time preceding enrollment, 130/397 (32.7%) received two doses, 69/397 (17.4%) received three doses and 197/397 (49.6%) children had not received the vaccine (Table 1). Strains of *S. pneumoniae* were isolated from 221 of the 397 healthy children surveyed, giving a total carrier rate of 55.7%, some of whom did receive the PCV7 vaccine and some not. The distribution of nasopharyngeal pneumococcal carrier rate among the West Bank districts is shown in Table 1.

**Table 1:** Distribution of nasopharyngeal pneumococcal carrier rates in children <2 years old from West Bank districts, including vaccinations and colonization rates (%).

District	One Dose	Two Doses	Three Doses	Not Vaccinated	Total	Positive *S. pneumoniae*	Colonization rate (%)	PCV7 serotypes
Bethlehem	1	12	-	-	13	7	53.8	0
Hebron	-	59	-	-	59	31	52.5	19F, 9V9A, 23F, 6B
Jericho	-	39	23	-	62	41	66.1	19F, 9V9A, 23F, 6B
Tubas	-	20	46	-	66	44	66.7	19F, 14, 23F, 6B
Ramallah	-	-	-	41	41	14	34.1	19F, 14, 23F, 6B
Jerusalem	-	-	-	15	15	7	46.6	23F, 6B, 14
Nablus	-	-	-	52	52	25	48.1	19F, 14, 6B
Tulkarem	-	-	-	24	24	13	54.2	19F, 23F, 6B
Qalqilia	-	-	-	17	17	9	52.9	19F, 23F
Jenin	-	-	-	39	39	23	58.9	23F, 4, 14, 6B
Salfeet	-	-	-	9	9	8	77.7	19F, 18 (A/B/C/F)
Total	1	130	69	197	397	221	55.7	55.7

The pneumococcal carriage rate among children of different ages was: 40.2% (33/87) in those 0-6 months; 62.5% (95/152) >6–12 months; 53.8% (56/104) >12–18 months and 64.8% (35/54) >18–24 months. The overall colonization rate among the PCV7-vaccinated children was 61.5% (123/200) (Table 1). The colonization rates among children who received either two or three doses of vaccine were 60.8% (79/130) and 63.8% (44/69), respectively, and among those unvaccinated 50.3% (99/197).

### Antimicrobial resistance

Susceptibility to antimicrobial agents was tested on 211 (95.5%) of the 221 isolates of *S. pneumoniae* (Table S1). Only 47/211 (22.3%) of them were fully susceptible to all the antibiotics tested, 58/211 (27.5%) were resistant to one of the antimicrobial agents, 34/211 (16.1%) were resistant to two, and 72/211 (34.1%) to more than two.

Of the 211 pneumococcal isolates examined, 23 (10.9%) were resistant to penicillin and 118 (55.9%) were intermediately resistant. Higher resistance rates were noted for trimethoprim-sulfamethoxazole, 97/211 (45.9%), and erythromycin, 64/213 (30.3%). None was resistant to cefotaxime and vancomycin.

Of the isolated bacterial strains of the serotypes 4, 6B, 9V, 14, 18C, 19F, and 23F found in the PCV7 vaccine that were examined for antimicrobial susceptibility, 59/79 (75%), 49/79 (62.0%) and 23/79 (29%) were non-susceptible to two, three and four antibiotics, respectively; whereas, of the bacterial strains of the additional serotypes found in the PCV13 vaccine, 14/38 (37%), 7/38 (18%) and 2/38 (5%) were not wholly or partially susceptible to two, three and four antibiotics, respectively (Table S1 and S2). Overall, the resistance of the isolates of *S. pneumoniae* to the antibacterial agents tested did not appear to be directly correlated with the childrens' vaccination status (p = 0.079). However, resistance of isolates to tetracycline and erythromycin did correlate significantly (p = 0.026 and 0.012, respectively) with whether they came from vaccinated and unvaccinated children. Isolates resistant to one or other of these two drugs were associated with the vaccinated group. In addition, percentage resistance of isolates of the serotypes in the PCV7 vaccine was higher (38/41 (92.7%), p<0.001) than that of isolates of the serotypes in the PCV13 vaccine (7.3%). Interestingly, resistance of isolates to tetracycline correlated highly with the serotypes of the PCV7 vaccine (39/42 (92.9%), p<0.001).

### Capsular Serotypes

Of the 221 isolates of *S. pneumoniae*, 191 (86.4%) were serotyped, leaving 30 (13.6%) untyped. However, the untyped isolates were sensitive to optochin and positive by the Real Time PCR-based method employing the *lytA* target gene. This showed they *were* isolates of *S. pneumoniae*. Isolates that did not typed with the test primers of the multiplex PCR were considered to be untypeable (NT). Altogether, 27 different serotypes were identified using the sequential multiplex PCR reactions [23].

The most common strains were, in decreasing order, of the serotypes: 6A, 19F, 23F, 6B, 14, 19A, 15B, 34 and 11A (Table 2). Strains of the serotypes 6A, 19F, 23F and 6B were the most prevalent (13.6%, 12.2%, 9.0% and 8.6%, respectively), followed by strains of the serotypes 19A and 14, (both at 4.1%) and 15B/15C and 34 (both at 3.6%). Among the carried isolates, 37.1% were of the serotypes in the PCV7 vaccine and 56.1% were those in the PCV13 vaccine (Table 2). Isolates carried by vaccinated children, were of the serotypes 19F (13%), 23F (6.5%), 6B (8.1%) and 14 (1.6%) in the PCV7 vaccine, in addition to which they also carried isolates of the serotypes 6A (13.0%) and 19A (5.7%) in the PCV13 vaccine. Those carried by non-vaccinated children were of exactly the same serotypes but in different percentages, 11.2%, 12.2%, 9.2%, 7.1%, 14.3% and 2.0%, respectively.

**Table 2:** Serotype frequencies of strains of *S. pneumoniae* isolated from nasopharyngeal samples and their distribution according to children' vaccination status.

Serotype	Total Number (%)	Vaccination status	
PCV7 serotypes		Vaccinated group	Unvaccinated group
19F	27 (12.2)	16 (13.0)	11 (11.2)
23F	20 (9.0)	8 (6.5)	12 (12.2)
6B	19 (8.6)	10 (8.1)	9 (9.2)
14	9 (4.1)	2 (1.6)	7 (7.1)
9V/9A*	4 (1.8)	4 (3.3)	0 (0)
4	2 (0.9)	0 (0)	2 (2.0)
18A/B/C/F*	1 (0.4)	0 (0)	1 (1.0)
Additional serotypes in PCV13			
6A	30 (13.6)	16 (13.0)	14 (14.3)
19A	9 (4.1)	7 (5.7)	2 (2.0)
3	2 (0.9)	1 (0.8)	1 (1.0)
1	1 (0.4)	0 (0)	1 (1.0)
Other vaccine Serotypes#			
15B/15C	8 (3.6)	5 (4.1)	3 (3.1)
11A/11D	7 (3.2)	4 (3.3)	3 (3.1)
10A	5 (2.3)	3 (2.4)	2 (2.0)
22F/22A	3 (1.4)	2 (1.6)	1 (1.0)
33F/33A/37	2 (0.9)	2 (1.6)	0 (0)
9N/9L	1 (0.4)	1 (0.8)	0 (0)
Other Serotypes			
34	8 (3.6)	7 (5.7)	1 (1.0)
15A/15F	5 (2.3)	3 (2.4)	2 (2.0)
21	5 (2.3)	3 (2.4)	2 (2.0)
35B	4 (1.8)	3 (2.4)	1 (1.0)
38/25F/25A	4 (1.8)	3 (2.4)	1 (1.0)
7C/7B/40	4 (1.8)	2 (1.6)	2 (2.0)
24 A/B/F	3 (1.4)	1 (0.8)	2 (2.0)
35F/47F	3 (1.4)	3 (3.1)	3 (3.1)
13	3 (1.4)	1 (1.0)	2 (2.0)
23A	2 (0.9)	0 (0)	2 (2.0)
Nontypeable (Not Determined)	30 (13.6)	19 (15.4)	11 (11.2)
Total	221 (100)	123 (55.7)	98 (44.3)

PCVs do not offer protection against all serotypes of serogroup 9 or 18.

^#^ Serotypes that were not covered in the protein conjugate vaccines, PCV7 and PCV13, but they are in the PCV23 vaccine.

Of the strains that were subjected to serotyping, 80.1% (113/141) were non-susceptible to penicillin. Of these, 118 were intermediately resistance and 23 were fully resistant. The common serotypes of the fully resistant strains were 19F, 6A, 23F, 19A, 6B and 24(A/B/F) while the common serotypes of the intermediately resistant strains were 6B, 19F, 6A, 23F, 14 and 15B/C (Table S1 and S2).

### Differentiation of Serogroup 6: 6A/C and 6B of *S. pneumoniae*

All serogroup 6 samples (49) were analyzed by RFLP, 28 of which were confirmed by sequencing, of which 25 were serotype 6A and 3 serotype 6B. The sequences obtained represent the 273 bp of the capsular gene region. The twenty-five 6A serotype sequences were all identical to the 6A serotype reference sequence (GenBank accession no. JF911497.1), while the three 6B serotype sequences were all identical to the 6B serotype reference sequence (GenBank accession no. JF911507.1), both of which are in the National Center for Biotechnology Information Database (http://www.ncbi.nih.gov/BLAST/). The two representative sequences found were deposited in the GenBank database (6A serotype accession no. KC834830 and 6B serotype KC834831).

As expected, fragment analysis, showed different patterns of digestion for the 2 serotypes. Of the 49 samples, 30 belonged to the serotype 6A/C and 19 to the serotype 6B. The results of samples analyzed by the RFLP and the DNA-sequencing procedures showed 100% agreement. Therefore, using the RFLP analysis developed here, it was possible to differentiate between the serotypes 6A/C and 6B, showing it to be a simple, rapid, direct and reliable method (Figure 1).

## Discussion

This is the first Palestinian national study documenting the prevalence of the serotypes of strains *S. pneumoniae* carried in the nasopharyngeal cavities of healthy children less than two years of age before the introduction of PCV as part of a Palestinian NIP. This study showed a carrier rate of 55.7%. This rate is similar to the 50% among Palestinian children from the Gaza strip reported by Regev-Yochay et al. [25]. In the neighboring region, Borer et al. [26] found a carrier rate of 58% among children aged < 5 years in day care centers in Southern Israel. Similar carrier rates were reported for Oman [27], Iran [28], Central Asia [29], the Netherland [30], and Kenya [31]. However, it was relatively higher compared to studies from other countries: Venezuela (28%) [32], Greece (29.4%) [33], Hong Kong (19.4%) [34], Italy (8.6%) [35], Europe as a whole region (21%) [36], the USA (20%) [12], Turkey (22.5%) [37] and Northern Taiwan (20.8%) [38].

Isolated strains of the serotypes 6A, a serotype of a strain in the PCV13 vaccine, 19F, 23F and 6B, serotypes of strains in the PCV7 vaccine, were most prevalent (13.6%, 12.2%, 9.0%, 8.6%, respectively), followed by strains of the serotypes: 19A and 14, (both 4.1%) and 15B/15C and 34 (both 3.6%). Worldwide prevalence studies on the nasopharyngeal carriage of *S. pneumoniae* have shown that, mostly, strains of the serotypes mentioned above are involved but with differences in percentages among them [12], [29], [36]. Of the strains carried by Palestinian children, 37.1% were of the serotypes covered by the PCV7 vaccine and 56.1% were those covered by the PCV13 vaccine. Clear difference was noticed between the vaccinated and unvaccinated groups regarding the serotypes 23F and 14 present in the PCV7 vaccine. Changes in pneumococcal serotypes were also observed in Portuguese vaccinated and unvaccinated populations [39]. While as would be expected, strains of the other serotypes existing in the PCV13 vaccine were found in equal percentages between vaccinated and unvaccinated children. This study adds to a previous Palestinian one [20] since it covers many geographical areas and a more in-depth analysis of the prevalence of strains of the serogroup 6. The previous study was restricted to a central region of Palestine and treated all their isolates of the serogroup 6 as a single entity. Furthermore, the study done here was done on healthy carriers. The incidence of pneumococcal disease is thought to be tied to the prevalence of asymptomatic carriers. Also, strains isolated from nasopharyngeal cavities can be used as determine serotype distribution and predict drug resistance. This, in turn, should improve the efficacy of treatment and vaccination.

The data presented here on the serotypes of carried strains, together with the data previously reported by Kattan et al. [20], provides Palestinian Health Authorities with strong scientific evidence to endorse ending using the PCV7 vaccine and substituting it with the PCV13 vaccine because of the extra strains serotypes it protects against, providing an additional 19.0% serotype coverage. Congruently, results obtained from a carriage study on healthy Israeli children aged below 36 months showed the added benefit of an extended serotype coverage by the PCV13 vaccine over the PCV7 vaccine of 21%, raising it from 46% to 67% [40]. Employing the PCV13 vaccine increases the cross-protective spectrum, at least against strains of the serotypes 6A, 19A, 3 and 1, which were also mentioned by Kattan et al., and are also responsible for IPD among Palestinian children [20]. The results from testing antimicrobial susceptibility of the strains of *S. pneumoniae* isolated from the Palestinian children in relation to the strain's serotypes, as mentioned above and discussed below, also support recommending using the PCV13 vaccine in the NIP in the West Bank.

For many years, penicillin has been the drug of choice against pneumococcal infections. However, microbial resistant has increased over past decades [12], [13], [41]. Here, resistance to penicillin was demonstrated with a non-susceptibility rate of 66.8% of the pneumococcal isolates examined (Table S2), which agrees other studies [26], [27], [34], [40]. As the prescribing and sale of antibiotics is largely locally unregulated, these rates are expected. The carriage of increasingly drug-resistant strains of *S. pneumoniae* by individuals could lead to the increased transmission of resistant pneumococcal disease at the community and national level. Also, these results should discourage physicians from using penicillin as empirical treatment for pneumococcal infections, particularly for invasive ones. In this study, the six most prevalent serotypes, 19F, 6A, 23F, 6B, 19A and 14, represent more than half (51.6%) of the pneumococcal strains encountered, and they are predominantly (80.8%) non-susceptible to penicillin. The recent study by Kattan et al. of children from the West Bank hospitalized with IPD caused by strains of *S. pneumoniae* identified 49 cases (40.8%) infected with strains of these serotypes [20]. Of all the serotypes covered by these two vaccines, the serotypes 19F, 6A, 23F, 6B, 19A, 14 and 9V are associated with the highest rate of penicillin non-susceptibility of the strains. Both vaccines, PCV7 and PCV13, contain components with some or all of these 7 serotypes, and 68 (48.2%) of the 141 penicillin resistant isolates possessed the serotypes in the PCV7 vaccine. An additional 18.4% of the 141 penicillin resistant isolates would accrue if the extra serotypes in the PCV13 vaccine were included. Regarding the examination of the efficacy of erythromycin, isolates of the serotypes 6B, 19F, 23F, 6A, 14, 19A, and 9V were associated with erythromycin resistant strains and the serotypes in the PCV7 and PCV13 vaccines cover 60 (54.5%) and 87 (79.0%), of the 110 erythromycin resistant isolates, respectively. Resistance to two antibiotics was observed in 75% and 37% of the isolates bearing the serotypes in the PCV7 and PCV13 vaccines, respectively.

In addition, there was a high degree of microbial resistance, against three and four antibacterial drugs by isolates with the serotypes in the PCV7 vaccine (Table S1). Resistance to tetracycline and erythromycin showed a significant correlation (p = 0.026 and 0.012, respectively) to vaccination status and bacterial isolates from the vaccinated group appeared to be more resistant to both drugs than isolates from the unvaccinated.

Serogroup 6 consists of strains of the serotypes 6A and 6B, and the more recently discovered serotypes 6C and 6D [10], [42], some of which are of importance in carriage and in causing invasive disease [43], [44]. Different molecular methods for sub-typing strains in serogroup 6 into the serotypes 6A, 6B, 6C and 6D have been described [24], [45]. However, one requires sophisticated equipment and training and one do not enable easy differentiation. Serological determination by the quellung reaction (capsular swelling) is considered the gold standard for serotyping. This method is limited as antisera are very expensive, good technical skill is required and interpretation of results is complicated. Here, a new PCR-RFLP method was developed and optimized that enabled the differentiation of the serotypes 6A/C and 6B. To our knowledge, no other RFLP assay for differentiating the serotypes 6A 6B and 6C has been developed and published. This procedure is simple, fast, reliable and less costly than sequencing, and can be used in any molecular laboratory.

## Conclusions

1. A high carrier rate of *S. pneumoniae* was observed in healthy Palestinian children.
2. The main strain serotypes were 19F, 23F, 14, 19A and, also, 6A/C and 6B that were differentiated using a new PCR-RFLP method developed and optimized in our laboratory.
3. High antibiotic resistance was revealed among the strains of *S. pneumoniae* isolated from nasopharyngeal cavities.
4. The high carrier rate of *S. pneumoniae* in the asymptomatic children combined with the results from testing antimicrobial susceptibility of the strains of *S. pneumoniae* isolated from their nasopharyngeal cavities, and the relationship of the isolates' serotypes compared with the serotypes in pneumococcal vaccines supports recommending using the PCV13 rather than the PCV7 vaccine in the NIP in the West Bank Region.

## Supporting Information

Table S1 Antibiotic susceptibility testing* results by serotype distribution.
(DOCX)

Table S2 **Antibiotics not susceptible (Both intermediate and resistant) to one or more antibiotics in regards to PCV Serotypes.**
(DOCX)

## References

[1] Brugger SD, Hathaway LJ, Muhlemann K (2009) Detection of Streptococcus pneumoniae strain cocolonization in the nasopharynx. J Clin Microbiol 47: 1750–1756. 19386843 10.1128/JCM.01877-08 PMC2691125

[2] Dagan R, Givon-Lavi N, Zamir O, Sikuler-Cohen M, Guy L, et al (2002) Reduction of nasopharyngeal carriage of Streptococcus pneumoniae after administration of a 9-valent pneumococcal conjugate vaccine to toddlers attending day care centers. J Infect Dis 185: 927–936. 11920317 10.1086/339525

[3] WHO (2007) Pneumococcal conjugate vaccine for childhood immunization—WHO position paper. Wkly Epidemiol Rec 82: 93–104. 17380597

[4] O'Brien KL, Wolfson LJ, Watt JP, Henkle E, Deloria-Knoll M, et al (2009) Burden of disease caused by Streptococcus pneumoniae in children younger than 5 years: global estimates. Lancet 374: 893–902. 19748398 10.1016/S0140-6736(09)61204-6

[5] Cardozo DM, Nascimento-Carvalho CM, Souza FR, Silva NM (2006) Nasopharyngeal colonization and penicillin resistance among pneumococcal strains: a worldwide 2004 update. Braz J Infect Dis 10: 293–304. 17293914 10.1590/s1413-86702006000400015

[6] Lin WJ, Lo WT, Chou CY, Chen YY, Tsai SY, et al (2006) Antimicrobial resistance patterns and serotype distribution of invasive Streptococcus pneumoniae isolates from children in Taiwan from 1999 to 2004. Diagn Microbiol Infect Dis 56: 189–196. 16725302 10.1016/j.diagmicrobio.2006.03.016

[7] Malfroot A, Verhaegen J, Dubru JM, Van Kerschaver E, Leyman S (2004) A cross-sectional survey of the prevalence of Streptococcus pneumoniae nasopharyngeal carriage in Belgian infants attending day care centres. Clin Microbiol Infect 10: 797–803. 15355410 10.1111/j.1198-743X.2004.00926.x

[8] Sjostrom K, Blomberg C, Fernebro J, Dagerhamn J, Morfeldt E, et al (2007) Clonal success of piliated penicillin nonsusceptible pneumococci. Proc Natl Acad Sci U S A 104: 12907–12912. 17644611 10.1073/pnas.0705589104 PMC1929012

[9] Arai S, Konda T, Wad A, Matsunaga Y, Okabe N, et al (2001) Use of antiserum-coated latex particles for serotyping Streptococcus pneumoniae. Microbiol Immunol 45: 159–162. 11293482 10.1111/j.1348-0421.2001.tb01284.x

[10] kamerling JP (2000) Streptococcus pneumoniae: molecular biology and mechanisms of disease. In: Tomasz A, editor. Pneumococcal polysaccharides: a chemical view. Larchmont, NY: Mary Ann Liebert. pp. 81–114.

[11] Melin M, Trzcinski K, Meri S, Kayhty H, Vakevainen M (2010) The capsular serotype of Streptococcus pneumoniae is more important than the genetic background for resistance to complement. Infect Immun 78: 5262–5270. 20855513 10.1128/IAI.00740-10 PMC2981297

[12] Hicks LA, Harrison LH, Flannery B, Hadler JL, Schaffner W, et al (2007) Incidence of pneumococcal disease due to non-pneumococcal conjugate vaccine (PCV7) serotypes in the United States during the era of widespread PCV7 vaccination, 1998-2004. J Infect Dis 196: 1346–1354. 17922399 10.1086/521626

[13] McIntosh ED, Reinert RR (2011) Global prevailing and emerging pediatric pneumococcal serotypes. Expert Rev Vaccines 10: 109–129. 21162625 10.1586/erv.10.145

[14] Harboe ZB, Valentiner-Branth P, Benfield TL, Christensen JJ, Hjuler T, et al (2008) Estimated effect of pneumococcal conjugate vaccination on invasive pneumococcal disease and associated mortality, Denmark 2000-2005. Vaccine 26: 3765–3771. 18513840 10.1016/j.vaccine.2008.04.040

[15] Kaltoft MS, Skov Sorensen UB, Slotved HC, Konradsen HB (2008) An easy method for detection of nasopharyngeal carriage of multiple Streptococcus pneumoniae serotypes. J Microbiol Methods 75: 540–544. 18801391 10.1016/j.mimet.2008.08.010

[16] Sahni V, Naus M, Hoang L, Tyrrell GJ, Martin I, et al (2012) The epidemiology of invasive pneumococcal disease in British Columbia following implementation of an infant immunization program: increases in herd immunity and replacement disease. Can J Public Health 103: 29–33. 22338325 10.1007/BF03404065 PMC6974085

[17] Aristegui J, Bernaola E, Pocheville I, Garcia C, Arranz L, et al (2007) Reduction in pediatric invasive pneumococcal disease in the Basque Country and Navarre, Spain, after introduction of the heptavalent pneumococcal conjugate vaccine. Eur J Clin Microbiol Infect Dis 26: 303–310. 17457623 10.1007/s10096-007-0294-4

[18] Feikin DR, Klugman KP (2002) Historical changes in pneumococcal serogroup distribution: implications for the era of pneumococcal conjugate vaccines. Clin Infect Dis 35: 547–555. 12173128 10.1086/341896

[19] Jacobs MR, Good CE, Bajaksouzian S, Windau AR (2008) Emergence of Streptococcus pneumoniae serotypes 19A, 6C, and 22F and serogroup 15 in Cleveland, Ohio, in relation to introduction of the protein-conjugated pneumococcal vaccine. Clin Infect Dis 47: 1388–1395. 18959493 10.1086/592972

[20] Kattan R, Abu Rayyan A, Zheiman I, Idkeidek S, Baraghithi S, et al (2011) Serotype distribution and drug resistance in Streptococcus pneumoniae, Palestinian Territories. Emerg Infect Dis 17: 94–96. 21192863 10.3201/eid1701.100886 PMC3204635

[21] McAvin JC, Reilly PA, Roudabush RM, Barnes WJ, Salmen A, et al (2001) Sensitive and specific method for rapid identification of Streptococcus pneumoniae using real-time fluorescence PCR. J Clin Microbiol 39: 3446–3451. 11574554 10.1128/JCM.39.10.3446-3451.2001 PMC88370

[22] CLSI (2012) Performance standards for antimicrobial susceptibility testing. M100–S22.

[23] Pai R, Gertz RE, Beall B (2006) Sequential multiplex PCR approach for determining capsular serotypes of Streptococcus pneumoniae isolates. J Clin Microbiol 44: 124–131. 16390959 10.1128/JCM.44.1.124-131.2006 PMC1351965

[24] Jin P, Xiao M, Kong F, Oftadeh S, Zhou F, et al (2009) Simple, accurate, serotype-specific PCR assay to differentiate Streptococcus pneumoniae serotypes 6A, 6B, and 6C. J Clin Microbiol 47: 2470–2474. 19535528 10.1128/JCM.00484-09 PMC2725661

[25] Regev-Yochay G, Abullaish I, Malley R, Shainberg B, Varon M, et al (2012) Streptococcus pneumoniae carriage in the Gaza strip. PLoS One 7: e35061. 22539955 10.1371/journal.pone.0035061 PMC3335158

[26] Borer A, Meirson H, Peled N, Porat N, Dagan R, et al (2001) Antibiotic-resistant pneumococci carried by young children do not appear to disseminate to adult members of a closed community. Clin Infect Dis 33: 436–444. 11462177 10.1086/321888

[27] Al-Yaqoubi MM, Elhag KM (2011) Serotype Prevalence and Penicillin-susceptibility of Streptococcus pneumoniae in Oman. Oman Med J 26: 43–47. 22043379 10.5001/omj.2011.11 PMC3191612

[28] Sanaei Dashti A, Abdinia B, Karimi A (2012) Nasopharyngeal carrier rate of Streptococcus pneumoniae in children: serotype distribution and antimicrobial resistance. Arch Iran Med 15: 500–503. 22827788

[29] Factor SH, LaClaire L, Bronsdon M, Suleymanova F, Altynbaeva G, et al (2005) Streptococcus pneumoniae and Haemophilus influenzae type B Carriage, Central Asia. Emerg Infect Dis 11: 1476–1479. 16229788 10.3201/eid1109.040798 PMC3310603

[30] Bogaert D, Engelen MN, Timmers-Reker AJ, Elzenaar KP, Peerbooms PG, et al (2001) Pneumococcal carriage in children in The Netherlands: a molecular epidemiological study. J Clin Microbiol 39: 3316–3320. 11526169 10.1128/JCM.39.9.3316-3320.2001 PMC88337

[31] Abdullahi O, Karani A, Tigoi CC, Mugo D, Kungu S, et al (2012) The prevalence and risk factors for pneumococcal colonization of the nasopharynx among children in Kilifi District, Kenya. PLoS One 7: e30787. 22363489 10.1371/journal.pone.0030787 PMC3282706

[32] Quintero B, Araque M, van der Gaast-de Jongh C, Escalona F, Correa M, et al (2011) Epidemiology of Streptococcus pneumoniae and Staphylococcus aureus colonization in healthy Venezuelan children. Eur J Clin Microbiol Infect Dis 30: 7–19. 20803226 10.1007/s10096-010-1044-6 PMC2998637

[33] Katsarolis I, Poulakou G, Analitis A, Matthaiopoulou I, Roilides E, et al (2009) Risk factors for nasopharyngeal carriage of drug-resistant Streptococcus pneumoniae: data from a nation-wide surveillance study in Greece. BMC Infect Dis 9: 120. 19640285 10.1186/1471-2334-9-120 PMC2724373

[34] Chiu SS, Ho PL, Chow FK, Yuen KY, Lau YL (2001) Nasopharyngeal carriage of antimicrobial-resistant Streptococcus pneumoniae among young children attending 79 kindergartens and day care centers in Hong Kong. Antimicrob Agents Chemother 45: 2765–2770. 11557466 10.1128/AAC.45.10.2765-2770.2001 PMC90728

[35] Marchisio P, Esposito S, Schito GC, Marchese A, Cavagna R, et al (2002) Nasopharyngeal carriage of Streptococcus pneumoniae in healthy children: implications for the use of heptavalent pneumococcal conjugate vaccine. Emerg Infect Dis 8: 479–484. 11996682 10.3201/eid0805.010235 PMC2732490

[36] Isaacman DJ, McIntosh ED, Reinert RR (2010) Burden of invasive pneumococcal disease and serotype distribution among Streptococcus pneumoniae isolates in young children in Europe: impact of the 7-valent pneumococcal conjugate vaccine and considerations for future conjugate vaccines. Int J Infect Dis 14: e197–209. 19700359 10.1016/j.ijid.2009.05.010

[37] Ozdemir B, Beyazova U, Camurdan AD, Sultan N, Ozkan S, et al (2008) Nasopharyngeal carriage of Streptococcus pneumoniae in healthy Turkish infants. J Infect 56: 332–339. 18377994 10.1016/j.jinf.2008.02.010

[38] Lo WT, Wang CC, Yu CM, Chu ML (2003) Rate of nasopharyngeal carriage, antimicrobial resistance and serotype of Streptococcus pneumoniae among children in northern Taiwan. J Microbiol Immunol Infect 36: 175–181. 14582561

[39] Sa-Leao R, Nunes S, Brito-Avo A, Frazao N, Simoes AS, et al (2009) Changes in pneumococcal serotypes and antibiotypes carried by vaccinated and unvaccinated day-care centre attendees in Portugal, a country with widespread use of the seven-valent pneumococcal conjugate vaccine. Clin Microbiol Infect 15: 1002–1007. 19392883 10.1111/j.1469-0691.2009.02775.x

[40] Shouval DS, Greenberg D, Givon-Lavi N, Porat N, Dagan R (2009) Serotype coverage of invasive and mucosal pneumococcal disease in Israeli children younger than 3 years by various pneumococcal conjugate vaccines. Pediatr Infect Dis J 28: 277–282. 19258924 10.1097/INF.0b013e31818e0e2e

[41] Scheifele D, Halperin S, Pelletier L, Talbot J, Lovgren M, et al (2001) Reduced susceptibility to penicillin among pneumococci causing invasive infection in children - Canada, 1991 to 1998. Can J Infect Dis 12: 241–246. 18159346 10.1155/2001/984958 PMC2094824

[42] Jin P, Kong F, Xiao M, Oftadeh S, Zhou F, et al (2009) First report of putative Streptococcus pneumoniae serotype 6D among nasopharyngeal isolates from Fijian children. J Infect Dis 200: 1375–1380. 19803727 10.1086/606118

[43] Hausdorff WP, Bryant J, Paradiso PR, Siber GR (2000) Which pneumococcal serogroups cause the most invasive disease: implications for conjugate vaccine formulation and use, part I. Clin Infect Dis 30: 100–121. 10619740 10.1086/313608

[44] Nahm MH, Lin J, Finkelstein JA, Pelton SI (2009) Increase in the prevalence of the newly discovered pneumococcal serotype 6C in the nasopharynx after introduction of pneumococcal conjugate vaccine. J Infect Dis 199: 320–325. 19099489 10.1086/596064 PMC2743180

[45] Pai R, Limor J, Beall B (2005) Use of pyrosequencing to differentiate Streptococcus pneumoniae serotypes 6A and 6B. J Clin Microbiol 43: 4820–4822. 16145148 10.1128/JCM.43.9.4820-4822.2005 PMC1234130

