# Supplementary material for: Streptococcus pneumoniae from Palestinian Nasopharyngeal Carriers: Serotype Distribution and Antimicrobial Resistance
Source: PLoS One. 2013 Dec 10;8(12):e82047. doi: 10.1371/journal.pone.0082047 (PMC3858295; doi:10.1371/journal.pone.0082047)
Supplement: Table S1 — Antibiotic susceptibility testing* results by serotype distribution. (DOCX) [file pone.0082047.s001.docx]

| **Serotype (n)** | **Antibiotics susceptibility** | | | | | | | | | | | |  | | | | | | | |
| --- | --- | --- | --- | --- | --- | --- | --- | --- | --- | --- | --- | --- | --- | --- | --- | --- | --- | --- | --- | --- |
|  | **Penicillin**  **n** | | | **Erythromycin**  **n** | | | **Tetracycline**  **n** | | | **Trimethoprime-Sulfamthoxanole n** | | | **Cefotaxime**  **n** | | | **Vancomycin**  **n** | | | | **Not Done**  **n** |
|  | **S** | **I** | **R** | **S** | **I** | **R** | **S** | **I** | **R** | **S** | **I** | **R** | **S** | **I** | **R** | **S** | **I** | | **R** |  |
| **PCV7 serotypes** | | | | | | | | | | | | | | | | | | | | |
| **19F (27)** | **2** | **17** | **6** | **8** | **4** | **13** | **15** | **7** | **3** | **3** | **2** | **20** | **25** | **0** | **0** | **25** | | **0** | **0** | **2** |
| **23F (20)** | **1** | **14** | **5** | **4** | **10** | **6** | **5** | **12** | **3** | **1** | **4** | **15** | **20** | **0** | **0** | **20** | | **0** | **0** | **0** |
| **6B (19)** | **1** | **17** | **1** | **1** | **2** | **16** | **6** | **10** | **3** | **3** | **0** | **16** | **19** | **0** | **0** | **19** | | **0** | **0** | **0** |
| **14 (9)** | **4** | **5** | **0** | **3** | **2** | **4** | **9** | **0** | **0** | **4** | **0** | **5** | **9** | **0** | **0** | **9** | | **0** | **0** | **0** |
| **9V/9A (4)** | **1** | **3** | **0** | **1** | **0** | **3** | **3** | **1** | **0** | **0** | **0** | **4** | **4** | **0** | **0** | **4** | | **0** | **0** | **0** |
| **4 (2)** | **2** | **0** | **0** | **2** | **0** | **0** | **2** | **0** | **0** | **1** | **1** | **0** | **2** | **0** | **0** | **2** | | **0** | **0** | **0** |
| **18 A/b/C/F (1)** | **0** | **0** | **0** | **0** | **0** | **0** | **0** | **0** | **0** | **0** | **0** | **0** | **0** | **0** | **0** | **0** | | **0** | **0** | **1** |
| **PCV 13 serotypes** | | | | | | | | | | | | | | | | | | | | |
| **6A (30)** | **8** | **14** | **5** | **5** | **12** | **10** | **25** | **1** | **1** | **20** | **4** | **3** | **27** | **0** | **0** | **27** | | **0** | **0** | **3** |
| **19A (9)** | **2** | **3** | **4** | **4** | **3** | **2** | **8** | **1** | **0** | **2** | **0** | **7** | **9** | **0** | **0** | **9** | | **0** | **0** | **0** |
| **3 (2)** | **1** | **0** | **0** | **1** | **0** | **0** | **1** | **0** | **0** | **1** | **0** | **0** | **1** | **0** | **0** | **1** | | **0** | **0** | **1** |
| **1 (1)** | **1** | **0** | **0** | **1** | **0** | **0** | **1** | **0** | **0** | **1** | **0** | **0** | **1** | **0** | **0** | **1** | | **0** | **0** | **0** |
| **Other Vaccine Serotypes** | | | | | | | | | | | | | | | | | | | | |
| **15B/15C (8)** | **2** | **5** | **0** | **1** | **4** | **2** | **5** | **2** | **0** | **2** | **0** | **5** | **7** | **0** | **0** | **7** | | **0** | **0** | **1** |
| **11A/11D (7)** | **5** | **2** | **0** | **6** | **0** | **1** | **6** | **1** | **0** | **4** | **1** | **2** | **7** | **0** | **0** | **7** | | **0** | **0** | **0** |
| **10A (5)** | **1** | **3** | **0** | **4** | **0** | **0** | **3** | **0** | **1** | **3** | **1** | **0** | **4** | **0** | **0** | **4** | | **0** | **0** | **1** |
| **22F/22A (3)** | **2** | **1** | **0** | **3** | **0** | **0** | **3** | **0** | **0** | **2** | **0** | **1** | **3** | **0** | **0** | **3** | | **0** | **0** | **0** |
| **33F/33A/37 (2)** | **1** | **1** | **0** | **1** | **1** | **0** | **2** | **0** | **0** | **0** | **0** | **2** | **2** | **0** | **0** | **2** | | **0** | **0** | **0** |
| **9N/9L (1)** | **1** | **0** | **0** | **1** | **0** | **0** | **1** | **0** | **0** | **0** | **1** | **0** | **1** | **0** | **0** | **1** | | **0** | **0** | **0** |
| **Other Serotypes** | | | | | | | | | | | | | | | | | | | | |
| **7C/7B/40 (4)** | **2** | **2** | **0** | **3** | **0** | **1** | **4** | **0** | **0** | **2** | **0** | **2** | **4** | **0** | **0** | **4** | | **0** | **0** | **0** |
| **34 (8)** | **5** | **3** | **0** | **6** | **2** | **0** | **8** | **0** | **0** | **8** | **0** | **0** | **8** | **0** | **0** | **8** | | **0** | **0** | **0** |
| **15A/15F (5)** | **2** | **3** | **0** | **1** | **0** | **4** | **1** | **2** | **2** | **3** | **0** | **2** | **5** | **0** | **0** | **5** | | **0** | **0** | **0** |
| **21 (5)** | **2** | **3** | **0** | **4** | **1** | **0** | **5** | **0** | **0** | **0** | **1** | **4** | **5** | **0** | **0** | **5** | | **0** | **0** | **0** |
| **35B (4)** | **1** | **3** | **0** | **3** | **1** | **0** | **4** | **0** | **0** | **4** | **0** | **0** | **4** | **0** | **0** | **4** | | **0** | **0** | **0** |
| **38/25F/25A (4)** | **4** | **0** | **0** | **3** | **0** | **1** | **3** | **1** | **0** | **3** | **0** | **1** | **4** | **0** | **0** | **4** | | **0** | **0** | **0** |
| **24 A/B/F (3)** | **2** | **0** | **1** | **3** | **0** | **0** | **3** | **0** | **0** | **2** | **0** | **1** | **3** | **0** | **0** | **3** | | **0** | **0** | **0** |
| **35F/47F (3)** | **1** | **2** | **0** | **3** | **0** | **0** | **3** | **0** | **0** | **3** | **0** | **0** | **3** | **0** | **0** | **3** | | **0** | **0** | **0** |
| **13 (3)** | **1** | **2** | **0** | **2** | **1** | **0** | **2** | **1** | **0** | **0** | **1** | **2** | **3** | **0** | **0** | **3** | | **0** | **0** | **0** |
| **23A (2)** | **1** | **1** | **0** | **2** | **0** | **0** | **2** | **0** | **0** | **2** | **0** | **0** | **2** | **0** | **0** | **2** | | **0** | **0** | **0** |
| **Nontypeable**  **(Not Determined) (30)** | **14** | **14** | **1** | **25** | **3** | **1** | **26** | **3** | **0** | **24** | **0** | **5** | **29** | **0** | **0** | **29** | | **0** | **0** | **1** |
| **Total** | **70** | **118** | **23** | **101** | **46** | **64** | **156** | **42** | **13** | **98** | **16** | **97** | **211** | **0** | **0** | **211** | | **0** | **0** | **10** |

Supporting Information files

Table S1: Antibiotic susceptibility testing* results by serotype distribution

*S, susceptible; I, intermediate; R, resistant. Antibiograms results were considered by disc diffusion method for all antibiotics except for Penicillin and Cefotaxime by E-test (MIC): Penicillin MIC (S≤0.06, I: 0.12-1, R≥2.0 μg/mL); Cefotaxime MIC (S≤1, I: 2, R≥4 μg/mL); Erythromycin disk diffusion (S≥21, I: 16–20, R≤15 mm); Tetracycline disk diffusion (S≥23, I: 19-22, R≤18 mm); Trimethoprime-Sulfamthoxanole disk diffusion (S≥19, I 16–18, R≤15 mm); Vancomycin disk diffusion (S≥17 mm).
