# Supplementary material for: Streptococcus pneumoniae from Palestinian Nasopharyngeal Carriers: Serotype Distribution and Antimicrobial Resistance
Source: PLoS One. 2013 Dec 10;8(12):e82047. doi: 10.1371/journal.pone.0082047 (PMC3858295; doi:10.1371/journal.pone.0082047)
Supplement: Table S2 — Antibiotics not susceptible (Both intermediate and resistant) to one or more antibiotics in regards to PCV Serotypes. (DOCX) [file pone.0082047.s002.docx]

**Table S2: Antibiotics not susceptible (Both intermediate and resistant) to one or more antibiotics in regards to PCV Serotypes**

| **Serotype** | **Susceptible to all antibiotics** | **Not susceptible to one antibiotic** | **Not susceptible to two ntibiotics** | **Not susceptible to three antibiotics** | **Not susceptible to four antibiotics** | **Not determined** | **Total** |
| --- | --- | --- | --- | --- | --- | --- | --- |
| **PCV7 Serotypes** | | | | | | | |
| 19 F | 2 | 1 | 5 | 7 | 10 | 2 | **27** |
| 23 F | 0 | 2 | 2 | 1 | 15 | 0 | **20** |
| 6B | 1 | 0 | 1 | 5 | 12 | 0 | **19** |
| 14 | 2 | 2 | 1 | 4 | 0 | 0 | **9** |
| 9 V/ 9 A | 0 | 1 | 0 | 2 | 1 | 0 | **4** |
| 4 | 1 | 1 | 0 | 0 | 0 | 0 | **2** |
| **PCV13 Serotypes** | | | | | | | |
| 6 A | 2 | 7 | 13 | 3 | 2 | 3 | **30** |
| 19 A | 1 | 2 | 1 | 4 | 1 | 0 | **9** |
| 3 | 1 | 0 | 0 | 0 | 0 | 1 | **2** |
| 1 | 1 | 0 | 0 | 0 | 0 | 0 | **1** |
| **Other serotypes not included within PCVs** | | | | | | | |
| 18 A/B/C/F | 0 | 0 | 0 | 0 | 0 | 1 | **1** |
| 15 B/ 15 C | 1 | 0 | 1 | 4 | 1 | 0 | **7** |
| 34 | 5 | 1 | 2 | 0 | 0 | 0 | **8** |
| 11 A/ 11 D | 2 | 3 | 2 | 0 | 0 | 0 | **7** |
| 15 A/ 15 F | 1 | 0 | 0 | 3 | 1 | 0 | **5** |
| 10 A | 1 | 1 | 2 | 0 | 0 | 1 | **5** |
| 21.00 | 0 | 2 | 2 | 1 | 0 | 0 | **5** |
| 35 B | 1 | 2 | 1 | 0 | 0 | 0 | **4** |
| 7C/7B/40 | 2 | 0 | 1 | 1 | 0 | 0 | **4** |
| 38/25F/25A | 3 | 0 | 0 | 1 | 0 | 0 | **4** |
| 22 F/ 22 A | 2 | 0 | 1 | 0 | 0 | 0 | **3** |
| 24 (A/B/F) | 2 | 0 | 1 | 0 | 0 | 0 | **3** |
| 35 F/ 47 F | 1 | 2 | 0 | 0 | 0 | 0 | **3** |
| 13 | 0 | 1 | 1 | 0 | 1 | 0 | **3** |
| 33 F/ 33 A/ 37 | 0 | 1 | 0 | 1 | 0 | 0 | **2** |
| 23 A | 1 | 1 | 0 | 0 | 0 | 0 | **2** |
| 9 N/ 9 L | 0 | 1 | 0 | 0 | 0 | 0 | **1** |
| UnTypable | 11 | 13 | 3 | 0 | 2 | 1 | **30** |
| **Total** | **54** | **44** | **42** | **37** | **46** | **10** | **221** |

**Note: Antibiotics tested included: Penicillin G, Erythromycin, Tetracycline, Trimethoprime-Sulfamthoxanole, Cefotaxime and Vancomycin**
